# Association between convenience stores near schools and obesity among school-aged children in Beijing, China

**DOI:** 10.1186/s12889-020-8257-0

**Published:** 2020-01-31

**Authors:** Shuang Zhou, Yu Cheng, Lan Cheng, Di Wang, Qin Li, Zheng Liu, Hai-Jun Wang

**Affiliations:** 1 Department of Maternal and Child Health, School of Public Health, Peking University, No.38 Xueyuan Rd, Beijing, 100191 China; 2 Leibniz Institute for Prevention Research and Epidemiology – BIPS, Bremen, Germany; 3 Faculty of Mathematics and Computer Science, University of Bremen, Bremen, Germany; 4 Reproductive Medicine Center, Peking University Third Hospital Beijing, Beijing, China

**Keywords:** Obesity, Convenience store, School, Children

## Abstract

**Background:**

Food environments have rapidly changed over the past years in China and children have more access to unhealthy food in convenience stores near schools. Since the studies on the association between convenience stores near schools and obesity had inconsistent results and no similar study in China, we conducted a study on the association in Beijing of China, which will provide scientific evidence for the intervention of childhood obesity.

**Methods:**

The study included 2201 students at grade 4 of 37 primary schools in Dongcheng or Miyun district of Beijing. The food environment data was acquired from AMAP, the free web-based geospatial service provider. The numbers of convenience stores were captured within the 800-m network buffer near schools using Geographic Information System. The weight and height of each student were measured by trained health professionals. Students’ dietary and physical behaviors and other information associated with obesity were collected with questionnaires for students and their parents. The generalized linear mixed model (GLMM) was used to analyze the data.

**Results:**

The average age of the students was 10.2 years (Standard Deviation (SD) = 0.33). The prevalence of obesity in students was 14.9%. The median number of convenience stores within the 800-m network buffer near schools was 24 in two districts. The number of convenience stores near each school varied from 5 to 67 (median: 25) in Dongcheng district and from 1 to 57 (median: 22) in Miyun district. After adjusting for the confounding factors at the family and individual levels, the association between convenience stores and childhood obesity was statistically significant. Additional ten convenience stores near schools were associated with an increased risk of obesity (Odds Ratio (OR) = 1.13, 95% confidence interval (*CI*): 1.03,1.24, *P* = 0.011). Compared with less than 24 convenience stores near schools, the students with more than or equal to 24 convenience stores near schools had an increased risk of obesity (OR = 1.49, 95% *CI*: 1.09, 2.03, *P* = 0.013).

**Conclusion:**

The students with more convenience stores near their schools had an increased risk of obesity. The findings provided evidence for developing public health policy to restrict the number of convenience stores near schools to prevent and control childhood obesity.

## Background

Childhood obesity has emerged to be a major public health problem. Based on the Chinese National Survey on Students Constitution and Health, the prevalence of overweight and obesity in Chinese children and adolescents has increased continuously since 1985, reaching 19.4% in 2014 [1]. Children with obesity have an increased risk of asthma [2], diabetes [3], evaluated blood pressure [4] and other cardiometabolic risk factors [5]. The adverse effects of childhood obesity can persist into adulthood, including increased risks of metabolic syndrome [6], cancer [7], non-alcoholic fatty liver [8], and cardiovascular mortality [9, 10].

Childhood obesity is a complex condition associated with the interaction of genetics and environmental factors which include physical activity, diet, and other relevant factors [11]. Recently policymakers and researchers have paid special attention to obesogenic environments [12]. A lot of previous studies have focused on the obesogenic effect of convenience stores [13]. The convenience stores near schools may be one of the obesogenic environments for students, because many unhealthy foods are sold in convenience stores, such as sugar-sweetened beverages and energy-dense snacks [14], and very limited healthy food are provided in convenience stores [15]. Especially, a previous study found that students aged 10 to 14 years old bought snacks mostly in convenience stores rather than other types of food stores [16]. Many school-aged children would like to buy unhealthy food such as sugar-sweetened beverages and energy-dense snacks on the way to school and from school [17], which may increase the intake of energy. Energy intake that exceeds energy expenditure will result in childhood obesity.

Because of the potential role of convenience stores near schools on students’ diet behaviors of the intake of fruits, vegetables, snacks, and fizzy drinks [18], some studies investigated the association of convenience stores near school and childhood obesity. However, there is no consensus on the role of convenience stores in childhood obesity. A few studies have demonstrated that convenience stores near schools increased the risk of children’s overweight or obesity [19–22], while others have shown an inverse association [23] or statistically insignificant association [24–32]. What’s more, the studies focusing on the association of convenience stores near school and children’s nutritional status were carried out in the USA [19, 21–23, 26, 28, 30], England [24, 27, 31], Canada [25, 26], and South Korea [29]. Only two studies investigated the association of convenience stores near school and children’s fatness in Chinese Taiwan [32, 33]. However, both of the studies were hampered by the limitation that they investigated the convenience stores in a circular buffer of schools without consideration of street networks [32, 33]. To our knowledge, there is no evidence on the association of convenience stores near school and obesity of school-aged children in mainland China. Since food environments and diet culture vary among different countries or regions [34, 35], it would be important to study its role in childhood obesity in mainland China.

The mainland of China has been facing rapid economic growth and dramatic change of food environments, and children have more access to unhealthy food in convenience stores near schools. Thus, we conducted the study aiming to elucidate the association of convenience stores near schools and obesity of school-aged children in Beijing of China, providing scientific evidence for the intervention of childhood obesity.

## Methods

### Study population

We recruited participants in grade 4 of 37 primary schools in Dongcheng or Miyun district of Beijing, China, from May to June in 2016. The inclusion criteria for schools were as follows: Firstly, non-boarding, non-specialty and non-minority schools. Secondly, schools with no less than 50 students in grade 4. Thirdly, schools’ leaders agreed to participate in the study. All the eligible primary schools (43 schools) which met the inclusion criteria were invited to complete the school-based obesity prevention survey. Finally, 37 of the 43 schools (86%) provided approval for the study, including 23 schools in Dongcheng district and 14 schools in Miyun district (Additional file 1: Fig. S1). We used the method of cluster sampling to recruit students in each school. All students in the 2–3 selected classes received the informed consent forms. We included the students whose parents agreed and signed the informed consent forms in the study. Exclusion criteria were as follows: Firstly, children with diseases of important organs, such as heart disease, hypertension, tuberculosis, asthma, hepatitis, and nephritis. Secondly, children with obesity caused by other reasons such as endocrine diseases, side effects of drugs and so on. Thirdly, children with dysplasia such as dwarfism and gigantism. Fourthly, children with body disability and malformation such as severe scoliosis, pigeon breast, lameness, Knock Knees, Bowlegs and so on. Finally, 2201 students of 37 primary schools were included in our study.

### Anthropometric measurements and definition of obesity

Height (cm) and weight (kg) were measured according to the standard procedure. Participants were required to wear light clothes and stand straight, barefoot and at ease when being measured. Weight was measured to the nearest 0.1 kg with a standardized lever scale and height to the nearest 0.1 cm with a portable stadiometer. Both the scales and stadiometers were calibrated before measurements. Body mass index (BMI) was calculated as body weight (kg) divided by height (m) squared (kg/m^2^). We used the standard of Working Group on Obesity in China (WGOC) to define the nutritional status [36]. Obesity was defined as a BMI at or above the 95th percentile for children of the same age and sex [36].

### Questionnaire investigation

We investigated students’ age, gender, physical activities, diet behaviors and other individual and family factors related to obesity with questionnaires for students and their parents. We collected students’ intake of vegetables, fruits, meat, milk, and sugar-sweetened beverages in the past seven days with the students’ questionnaires. We designed the questions of the students’ diet behaviors according to the Block Kids Food Screener (BKFS). The validity study showed that the correlation between BKFS and three 24-h dietary recalls was 0.526, 0.600, 0.681, 0.478, 0.869 in the intake of vegetables, fruits, meat, sugar-sweetened beverages, and milk, respectively [37]. We asked the students about the frequency and amount of the foods and beverages consumed in the past seven days. For example, the following questions “How many days do you eat vegetables in the past seven days” and “How many portions of vegetables do you eat on average per day” were asked. The frequency was from zero to seven days and the amount was half of one portion, one portion, two portions, and more than or equal to three portions. One portion was 150 g for vegetables and fruits, 80–100 g for meat, and 250 ml for sugar-sweetened beverages and milk. According to the Dietary Approaches to Stop Hypertension (DASH) [38], in the Integrated Guidelines for Cardiovascular Health and Risk Reduction in Children and Adolescents, we adopted the following evaluation criteria. The recommended intakes of vegetables and fruits were more than 3 portions and 2 portions per day, respectively. The recommendation of meat intake was less than 2 portions per day for boys and 1 portion per day for girls. The recommendation of milk was more than 2 portions per day. The recommended intake of sugar-sweetened beverages was less than 3 portions per week. If the intake of vegetables, fruits, and milk reached or exceeded the recommendation, the score was 1, else, the score was the actual intake divided by the recommended amount. If the intakes of meat and sugar-sweetened beverages were within the recommendation, the score was 1, else, the score was 1- (actual intake- recommended amount) / recommended amount, and if the score was negative, we recorded the score as zero. We calculated the score of the dietary pattern as the sum of all the above scores. The summed score ranged from zero to five and the higher score reflected greater adherence to the recommended dietary intakes. We investigated the physical behaviors according to the American Youth Risk Behavior Survey Questionnaire [39], and physical activity recommendation was that children had moderate or vigorous physical activities for more than or equal to one hour every day [40]. According to the guideline of screen time for children and adolescents in the American Academy of Pediatrics, the recommendation of screen time was less than two hours every day [41]. We collected the information about parents’ height, weight, and education, and family type (family with only one child or with more than one child) with the questionnaire for parents.

### Density of convenience stores

We used AMAP, the free web-based geospatial service provider, to geocode and locate the schools’ addresses. By using the Application Programming Interface (API) on the AMAP open platform, we accessed to Point of Interest data (POI). Convenience stories were defined as the POI from 060200 to 060202 in AMAP, which included all small convenience stores and grocery stores such as 7-Eleven, Good Neighbor, Lin Jia, and other chain and non-chain convenience stores. We calculated the density of convenience stores located within an 800-m street network buffer of school centroid. We used the 800-m buffer for the reason that it approximately corresponded to 10- min walk, which was commonly used in previous studies [13].

We also undertook an on-foot ground audit in 8 schools. We recorded the actual information of the convenience stores and compared the differences in the information between the AMAP and the ground audit. We also calculated the accuracy of the convenience stores from the map, by the number from both map and ground audit divided by the number from the ground audit. Finally, the accuracy for the convenience stores was 80.9%, which was similar to the previous study [42].

### Statistical analyses

Means and standard deviations were calculated for continuous variables with normal distribution, while medians and interquartile ranges for non-normally distributed variables. Frequencies were calculated for categorical variables. We defined more than or equal to the median number of convenience stores as more convenience stores and less than the median number as fewer convenience stores. We tested differences between Dongcheng district and Miyun district in categorical characteristics with the chi-square test, normally distributed continuous characteristics with t test, non-normally distributed continuous characteristics with Wilcoxon signed-rank test.

We used Generalized Linear Mixed Model to explore the association of childhood obesity and convenience stores near schools. We used childhood obesity(obesity = 1, non-obesity = 0) as the outcome variable and the number of convenience stores as the continuous predictor or more convenience stores as the categorical predictor. PROC GLIMMIX procedure was run with schools modeled as a random effect because of the cluster sampling of schools, using logit as the link function. Based on the previous studies on the association between food environment and childhood obesity [13], we chose the district level, family level, and individual level covariates and adjusted the covariates in the order of district, family, and individual level. First, we used the null model (model 1), not controlling for covariates, to examine the association of childhood obesity and convenience stores. Secondly, we used model 2 to adjust for the district. Model 3 further included the family confounding factors, i.e. whether the parents were obese, parents’ education levels, and family type.Then model 4 further adjusted for age and gender of children. Finally, in the full model 5, we further adjusted for dietary and physical behaviors of children that involved the score of dietary pattern, meeting the recommendation of moderate or vigorous physical activities or not, and meeting the recommendation of screen time or not. The model was built as follows:

$$ {\displaystyle \begin{array}{l}\mathrm{logit}\left(P\left({y}_{ij}= obesity|{x}_{ij}\right)\right)\\ {}={\beta}_0+{\mu}_{0j}+{\beta}_{1j}\times {\mathrm{school}}_j+{\beta}_2\times {x}_{convenience\ storesij}+{\beta}_3{x}_{3 ij}+\cdots +{\beta}_p{x}_{pij}+\upvarepsilon \end{array}} $$

*β*_*0*_: the fixed intercept.

*μ*_0*j*_: the random intercept.

i: individual level.

j: school level.

*β*_1*j*_ × school_*j*_: the random effect of the school.

convenience stores: either as a continuous variable or a categorical variable.

ε: the residual effect.

All statistical analyses were performed using SAS 9.4.

## Results

### General characteristics

The 2201 students in grade 4 of 37 schools participated in the study. The characteristics of students were shown in Table 1. The average age was 10.2 years (SD = 0.33). 50.3% of the students were boys. The prevalence of obesity in students was 14.9%. Students in Dongcheng district were younger than those in Miyun District. More students in Miyun district had siblings, had obese parents and had parents with low education than those in Dongcheng district.

**Table 1:** General characteristics of participants and convenience stores in the study

Variables	All	Dongcheng District	Miyun District	*P*^a^
Age, year (mean ± SD)	10.2 ± 0.33	10.2 ± 0.33	10.3 ± 0.33	0.017^b^
Gender (%)				0.423^c^
Boy	1107 (50.3)	633 (49.6)	474 (51.3)	
Girl	1094 (49.7)	644 (50.4)	450 (48.7)	
Obesity (%)				0.618^c^
Yes	329 (14.9)	195 (15.3)	134 (14.5)	
No	1872 (85.1)	1082 (84.7)	790 (85.5)	
Family type (%)				< 0.001^c^
with only one child	1595 (73.2)	1014 (80.2)	581 (63.6)	
with more than one child	584 (26.8)	251 (19.8)	333 (36.4)	
Father’s Obesity (%)				< 0.001^c^
Yes	381 (17.7)	192 (15.3)	189 (21.1)	
No	1767 (82.3)	1060 (84.7)	707 (78.9)	
Mother’s Obesity (%)				< 0.001^c^
Yes	149 (6.9)	59 (4.7)	90 (10.0)	
No	2002 (93.1)	1190 (95.3)	812 (90.0)	
Father’s Education (%)				< 0.001^c^
Primary school or below	59 (2.7)	13 (1.0)	46 (5.1)	
Junior high school	338 (15.7)	72 (5.8)	266 (29.7)	
High school or Technical secondary school	461 (21.5)	159 (12.7)	302 (33.7)	
Junior college or Vocational college	400 (18.6)	253 (20.2)	147 (16.4)	
University degree or above	891 (41.5)	755 (60.3)	136 (15.2)	
Mother’s Education (%)				< 0.001^c^
Primary school or below	74 (3.4)	23 (1.8)	51 (5.7)	
Junior high school	376 (17.5)	81 (6.5)	295 (32.8)	
High school or Technical secondary school	435 (20.2)	166 (13.3)	269 (29.9)	
Junior college or Vocational college	423 (19.7)	279 (22.3)	144 (16.0)	
University degree or above	843 (39.2)	702 (56.1)	141 (15.7)	
Number of convenience stores within 800 m network buffer near school				0.597^d^
Median	24	25	22	
Interquartile range	(14, 43)	(15, 38)	(3, 45)	
Range	(1, 67)	(5, 67)	(1, 57)	

^a^ *P* value for differences in general characteristics between Dongcheng district and Miyun district

^b^ Difference was tested using t test

^c^ Difference was tested using chi-square test

^d^ Difference was tested using Wilcoxon signed-rank test

### Dietary and physical behaviors

Table 2 showed the dietary and physical behaviors of the students. The average score of the dietary pattern was 3.5 (SD = 0.78). Only the average consumption of meat and sugar-sweetened beverages met the recommendation, while the intakes of vegetables, fruits, and milk were all less than the recommendation. Only 15.5% of students met the recommendation of moderate or vigorous physical activities. And 81.1% of students met the recommendation of screen time of less than 2 h every day. Students in Dongcheng district had a higher sum of the dietary score than those in Miyun district. Students in Dongcheng district ate more vegetables, fruits, meat, and milk, and less sugar-sweetened beverages than those in Miyun district.

**Table 2:** The dietary and physical behaviors among students in the study, mean ± SD or N (%)

Variables	All	Dongcheng District	Miyun District	*P* value^a^
Diet score (mean ± SD)	3.5 ± 0.78	3.6 ± 0.75	3.3 ± 0.80	< 0.001^b^
Vegetable Intake, portion/day (mean ± SD)	1.5 ± 0.90	1.6 ± 0.91	1.4 ± 0.87	< 0.001^b^
Fruit Intake, portion /day (mean ± SD)	1.5 ± 0.96	1.6 ± 0.96	1.3 ± 0.93	< 0.001^b^
Meat Intake, portion /day (mean ± SD)	0.7 ± 0.69	0.8 ± 0.74	0.5 ± 0.59	< 0.001^b^
SSB Intake, portion /week (mean ± SD)	1.9 ± 3.16	1.5 ± 2.67	2.4 ± 3.66	< 0.001^b^
Milk Intake, portion /day (mean ± SD)	1.1 ± 0.89	1.2 ± 0.91	1.0 ± 0.86	< 0.001^b^
Meeting the Recommendation of MVPA (%)				0.208^c^
Yes	339 (15.5)	186 (14.7)	153 (16.7)	
No	1845 (84.5)	1080 (85.3)	765 (83.3)	
Meeting the Recommendation of Screen time (%)				< 0.001^c^
Yes	1761 (81.1)	1095 (86.7)	666 (73.3)	
No	411 (18.9)	168 (13.3)	243 (26.7)	

^a^ *P* value for differences in general characteristics between Dongcheng district and Miyun district

^b^ Difference was tested using t test

^c^ Difference was tested using chi-square test

SSB, sugar sweetened beverages

MVPA, moderate or vigorous physical activities

The recommendation of MVPA was equal to or more than one hour every day

The recommendation of screen time was less than 2 h every day

### Convenience stores near school

The number of convenience stores near schools was shown in Table 1. There was no statistically significant difference in the number of convenience stores within the 800 m network buffer near schools between the two districts. The median number of convenience stores within the 800-m network buffer near schools was 24 in two districts. The median numbers of convenience stores within the 800-m network buffer near schools in Dongcheng district and Miyun district were 25 and 22, respectively. There was marked variation in the number of convenience stores within the 800-m network buffer near each school, ranging from 5 to 67 in Dongcheng district and ranging from 1 to 57 in Miyun district.

### Association between the number of convenience stores near school and childhood obesity

In the analysis of the generalized linear mixed model (Table 3), we adjusted for the confounding factors at district, family, and individual levels gradually. When we adjusted for the family confounding factors in Model 3, the association between convenience stores and childhood obesity became significant. When we further adjusted for the individual confounding factors, the risk of childhood obesity increased gradually. In the full model, there was a significant association between convenience stores near school and childhood obesity. We found that additional ten convenience stores near schools were associated with an increased risk of obesity (OR = 1.13, 95% *CI*: 1.03,1.24). After dividing the number of convenience stores according to the median, the association was more significant. Compared with fewer convenience stores near schools (less than 24), students with more convenience stores near schools (more than or equal to 24) had an increased risk of obesity (OR = 1.49, 95% *CI*: 1.09, 2.03).

**Table 3:** Association between convenience stores within the 800 m network buffer near school and students’ obesity in two districts

	Continuous variable (ten additional convenience stores)			Categorical variable^a^ (more convenience stores vs less convenience stores)		
	OR^b^	95% CI	*P* value	OR^c^	95% CI	*P* value
Model 1	1.06	(0.97, 1.15)	0.178	1.17	(0.89, 1.54)	0.259
Model 2	1.06	(0.97, 1.15)	0.194	1.16	(0.88, 1.54)	0.291
Model 3	1.09	(1.01, 1.18)	0.030	1.34	(1.02, 1.76)	0.035
Model 4	1.10	(1.01, 1.19)	0.029	1.37	(1.04, 1.81)	0.026
Model 5	1.13	(1.03, 1.24)	0.011	1.49	(1.09, 2.03)	0.013

^a^Categorical variable was divided by 24: the median number of convenience stores

^b^OR for continuous variable was the odds ratio of ten additional convenience stores

^c^OR for categorical variable was more than or equal to the median number of 24 versus less than 24 (reference)

Model 1: null model

Model 2: adjusted for districts

Model 3: Model 2 + father’s obesity, mother’s obesity, family type, father’s education, and mother’s education

Model 4: Model 3 + age, gender

Model 5: Model 4 + diet score, meeting the recommendation of MVPA, and meeting the recommendation of screen time

## Discussion

We found a significant association between convenience stores near school and childhood obesity in our study. Additional ten convenience stores near schools were associated with an increased risk of obesity (OR = 1.13, 95% *CI*: 1.03,1.24). Compared with less than 24 convenience stores near schools, the students with more than or equal to 24 convenience stores near schools had an increased risk of obesity (OR = 1.49, 95% *CI*: 1.09, 2.03).

Our findings of a positive association between convenience stores near schools and the prevalence of obesity corresponded to some studies carried out in North America. Sanchez *et.al* [19] found that students with each additional convenience store near schools had an estimated 1% higher prevalence of overweight. Howard *et.al* [21] and Langellier *et.al* [22] showed that the presence of a convenience store within an 800-m buffer was associated with 1.2 and 1.6% higher prevalence of overweight compared with those schools without a convenience store nearby, respectively.

Several studies in western countries were not in accordance with our study, which couldn’t identify a significant association between convenience stores near school and childhood obesity. The inconsistency may be due to the differences in types of the buffer zone, buffer distance, source of information about food environments, definitions of food environments, measurements of participants’ weight and height, adjustment of the potential covariates of dietary and physical behaviors, and sample size. Some of the studies investigated the food environments in a circular buffer instead of a street network buffer [25–27, 29]. However, researches showed it was better to detect the food environments near schools within a road network buffer than in a circular buffer [43]. The difference in buffer distance may also result in inconsistency. An 800-m buffer approximately corresponded to a 10-min walk [13], while a 500-m or 1000-m buffer meant a walk for a shorter time or a longer time, respectively. The type of food bought by students in convenience stores within different distances may be different. The source of information about food environments and their definitions had an impact on the association between the number of convenience stores near school and childhood obesity. Precise information from a reliable source was important for the study. Similarly, the accuracy of the weight and height of children was also important, while some studies obtained the information of weight and height by self-report [25, 26, 28]. Physical activities and diet were strongly associated with obesity, which should be controlled in the statistical models. As shown in our results, the risk of childhood obesity increased when we adjusted for the individual dietary and physical behaviors. But some studies were lack of the individual information of dietary or physical behaviors [30, 31]. Since countries with different diet culture sold varied kinds of foods, in other words, children in Beijing of China might have access to different foods compared with foreign children, so it is difficult to compare the results from studies in other countries or regions with that of our study.

In China, there was no study on the association between convenience stores near school and childhood obesity in mainland, and only two studies were conducted in Chinese Taiwan [32, 33]. The two studies didn’t find a significant association between convenience stores near school and childhood obesity. The reason why they were inconsistent with our study may be that they investigated the convenience store in a circular buffer of schools without consideration of street network and the distance was 500 m and 1000 m, which were different from our study [32, 33].

Studies have also shown that school-aged children would like to buy sugar-sweetened beverages and energy-dense snacks when they went to school or went back home [17]. Intake of unhealthy food will increase the risk of childhood obesity for that the energy intake exceeds the energy expenditure [44]. It could explain the number of convenience stores near schools is associated with an increased risk of childhood obesity.

Our study had the significance not only for parents but also for policymakers. Although China has announced the policy that schools are banned to have retail shops and vending machines within the campus, the significant role of the convenience stores near the campus in childhood obesity was ignored. The findings provided evidence for developing the public health policy to restrict the number of convenience stores near schools to prevent and control childhood obesity.

There are some strengths in our study. First, to our knowledge, it was the first study using the geographical information system to investigate the association between convenience stores near schools and childhood obesity in mainland of China. Besides, we included information at the district, family, and individual levels, which allowed us to adjust for the potential covariates at different levels. Especially, we adjusted for the dietary and physical behaviors, which were the important influencing factors of obesity, to assess the true association between convenience stores near schools and childhood obesity.

There are some limitations in the present study. Firstly, given the cross-sectional design of our study, it has the limited capability to infer causality between convenience stores near school and childhood obesity. Secondly, although we adjusted for several confounding factors at the district, family, and individual levels, we can’t rule out the possibility of residual confounding factors. We didn’t adjust for other food environments like the density of fast food outlets, supermarkets or other markets in the region and vending machines in the school. However, according to the situation in China, the fast food shop serves more expensive foods than convenience stores, and students are unlikely to go to supermarkets or other markets alone. The Education Committee in Beijing has banned the retail shops and vending machines on the campus since 2013, so there’re no retail shops within our study schools. So our study results indicated convenience stores near schools had a more important role in primary students in China with little potential for the residual confounding of other types of food stores, which need further studies for validation. What’s more, our study was conducted in the two districts of Beijing. Further studies are needed to investigate the association between convenience stores near schools and childhood obesity in a large number of Chinese schools in different regions of China.

## Conclusion

The students with more convenience stores near their schools had an increased risk of obesity. The findings provided evidence for developing the public health policy to restrict the number of convenience stores near schools to prevent and control childhood obesity.

## Supplementary information

**Additional file 1: Figure S1.** Flowchart of primary schools in the study

## Data Availability

The datasets used and analyzed during the current study are available from the corresponding author on reasonable request.

## Abbreviations

API: Application Programming Interface
BKFS: Block Kids Food Screener
BMI: Body mass index
CI: Confidence Interval
DASH: Dietary Approaches to Stop Hypertension
GLMM: Generalized linear mixed model
OR: Odds Ratio
POI: Point of Interest data
SD: Standard Deviation
WGOC: Working Group on Obesity in China
