# Supplementary figures and images for: Association between convenience stores near schools and obesity among school-aged children in Beijing, China
Source: BMC Public Health. 2020 Jan 31;20:150. doi: 10.1186/s12889-020-8257-0 (PMC6995088; doi:10.1186/s12889-020-8257-0)

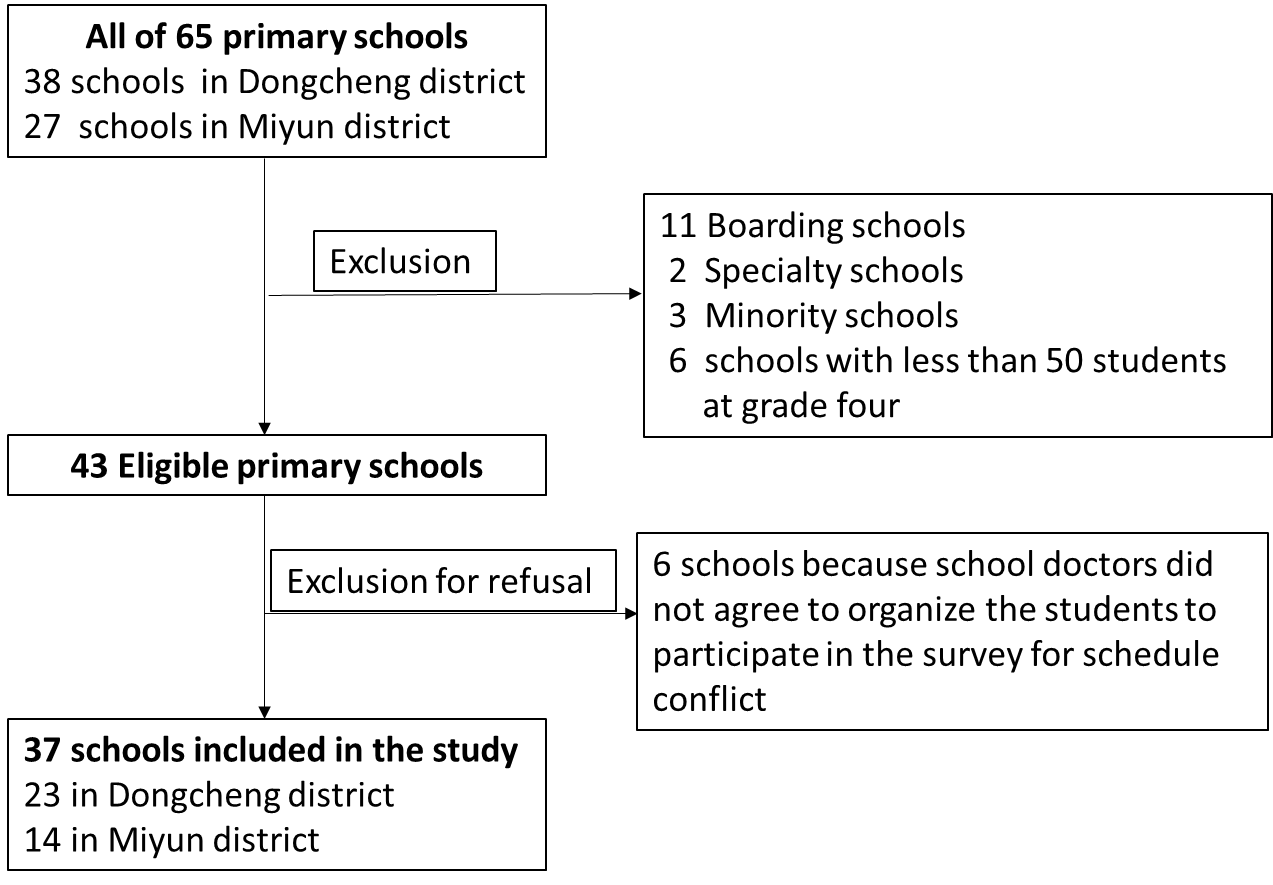


**Figure S1 Flowchart of primary schools in the study**

Supplement: Supplementary file 1 — Additional file 1: Figure S1. Flowchart of primary schools in the study [file 12889_2020_8257_MOESM1_ESM.docx]
